# Extended indications for sperm retrieval: summary of current literature

**DOI:** 10.12688/f1000research.20564.1

**Published:** 2019-12-04

**Authors:** Sandro C. Esteves, Matheus Roque

**Affiliations:** 1 ANDROFERT, Andrology and Human Reproduction Clinic, Referral Center for Male Reproduction, Campinas, SP, Brazil; 2 Department of Surgery (Division of Urology), University of Campinas (UNICAMP), Campinas, SP, Brazil; 3 Faculty of Health, Aarhus University, Aarhus, Denmark; 4 MATER PRIME, Reproductive Medicine, São Paulo, SP, Brazil

**Keywords:** sperm DNA fragmentation, sperm chromatin damage, sperm retrieval, testicular sperm, ejaculated sperm, assisted reproductive technology, in vitro fertilization, intracytoplasmic sperm injection, oligozoospermia, cryptozoospermia, pregnancy, offspring health, male infertility

## Abstract

Sperm retrieval combined with intracytoplasmic sperm injection (ICSI) is the treatment of choice for couples with untreatable azoospermia-related infertility. However, an increasing body of evidence has been mounting, suggesting that ICSI with testicular sperm instead of ejaculated sperm (when both are available) increases pregnancy outcomes in some specific scenarios. This has led to the exploration of extended indications for sperm retrieval. This review summarizes the current literature concerning sperm retrieval and ICSI for non-azoospermic men with elevated sperm DNA fragmentation, oligozoospermia, and cryptozoospermia.

## Introduction

Intracytoplasmic sperm injection (ICSI) was an extraordinary achievement in the field of assisted reproduction technology (ART). Introduced in 1992 as a modification of conventional *in vitro* fertilization (IVF), ICSI enables men with low sperm quantity and quality to father a child ^1, 2^. Nowadays, ICSI has become not only the most commonly used method of fertilization in ART but also the method of choice for overcoming untreatable severe male factor infertility ^3^.

ICSI is typically carried out with ejaculated sperm, which are generally regarded as having the highest fertilization potential since they have completed their transit through the male reproductive tract. By contrast, sperm retrieval methods—developed a few years after the introduction of ICSI—have been used to harvest sperm from the epididymides and testes of men with azoospermia-related infertility ^4, 5^. After retrieval of epididymal or testicular sperm, ICSI is mandatory as the retrieved gametes are unable to fertilize the oocytes by conventional IVF.

However, as experience accumulated, reports of an association between semen quality and ICSI outcomes increased steadily ^6– 8^. Concerns of a possible role of the paternal gamete on ICSI outcomes led Greco *et al.*, in 2005, to investigate the utility of sperm retrieval in a group of 18 non-azoospermic patients with elevated sperm DNA fragmentation (SDF) on neat semen ^9^. On the day of oocyte retrieval, the male partners underwent sperm retrieval using percutaneous or open methods to harvest sperm from the seminiferous tubules. In this series, ICSI with testicular sperm (Testi-ICSI) resulted in eight clinical pregnancies (44.5%) whereas only one pregnancy (5.6%) that ended in miscarriage had been obtained in previous ICSI cycles with the use of ejaculated sperm.

Given this information, the utility of sperm retrieval in indications other than azoospermia has been investigated. Here, the current support for these indications, including elevated SDF, severe oligozoospermia, and cryptozoospermia—denoted by very few spermatozoa (or none) in the fresh ejaculate but observed after microscopic examination of centrifuged pellet—will be summarized.

## Extended sperm retrieval indications: biological plausibility

It is well established that sperm chromatin integrity is vital for the birth of healthy infants ^10^. Fertilization of oocytes by sperm with DNA fragmentation might increase the risk of fertilization failure, embryo development arrest, implantation failure, miscarriage, congenital malformations, and perinatal and postnatal morbidity ^11– 13^. Notably, infertile men often have elevated SDF rates in neat semen ^14, 15^. Varicocele, systemic diseases, male accessory gland infections, advanced paternal age, obesity, lifestyle and environmental factors, radiation, and heat exposure are some of the conditions associated with SDF ^16, 17^. These stressors have in common the trait of oxidative stress, which represents a significant cause of SDF ^18^. The mechanisms involve reactive oxygen species (ROS) attack on sperm membranes and nuclear and mitochondrial DNA, mostly during sperm transit through the male reproductive tract ^19– 21^.

Interestingly, data from human studies assessing paired testicular and ejaculated specimens of non-azoospermic men indicate that SDF is two to three times lower in testicular sperm than in ejaculated sperm ^9, 22– 25^. A 2017 systematic review—followed by a meta-analysis—compiled the results of five studies including 143 patients and showed that the mean difference (MD) in SDF rates was −24.6% (95% confidence interval [CI] −32.5 to −16.6%, I ^2^ = 92%, *P* <0.001) in favor of testicular sperm ^26^. In that report, SDF was measured by using the terminal deoxyribonucleotide transferase–mediated dUTP nick-end labeling (TUNEL) assay (four studies, pooled MD: −19.8%, 95% CI −22.3 to −17.2%, I ^2^ = 15%, *P* <0.001) or the sperm chromatin dispersion (SCD) assay (one study, MD: −32.4%, 95% CI −34.85 to −29.95%, *P* <0.001).

## Elevated sperm DNA fragmentation

After the report by Greco *et al.* ^9^, several authors investigated the utility of sperm retrieval in non-azoospermic men with elevated SDF in neat semen ( Table 1) ^19, 24, 25, 27– 32^. In a 2017 systematic review, we aggregated the evidence of five studies including 507 ICSI cycles ^26^. In total, 3,840 oocytes were injected with either ejaculated sperm or testicular sperm. Using meta-analysis, we showed higher clinical pregnancy rates (odds ratio [OR] 2.42, 95% CI 1.57 to 3.73, I ^2^ = 34%, *P* <0.0001) and live birth rates (OR 2.58, 95% CI 1.54 to 4.35, I ^2^ = 0%, *P* = 0.0003), and lower miscarriage rates (OR 0.28, 95% CI 0.11 to 0.68, I ^2^ = 11%, *P* = 0.005) when comparing Testi-ICSI with ejaculated ICSI.

**Table 1.** Studies reporting ICSI outcomes with testicular versus ejaculated sperm in non-azoospermic men with high sperm DNA fragmentation in the neat semen.

Study characteristics		Indication			Sperm retrieval method		Outcomes		
Author (year)	Design	Subjects and cohort size (N)	Test used for sperm chromatin damage assessment and cutoff values (%)	Paired SDF results in testicular and ejaculated sperm (%)	Sperm retrieval method	Sperm retrieval success and complication rates (%)	Fertilization rate (%)	Clinical pregnancy rate (%)	Ongoing pregnancy rate or live birth rate ^a^ (%)
Greco *et al*. ^9^ (2005)	Case series	Predominantly normozoospermic infertile men (18); couples with history of ICSI failure performed with ejaculated sperm	TUNEL (15)	23.6 ± 5.1 (E) and 4.8 ± 3.6 (T) ( *P* <0.001)	TESE and TESA	100.0 and NR	74.9 ^b^	44.4 ^c^	NR
Sakkas and Alvarez ^19^ (2010)	Case series	Couples with history of IVF/ICSI failure (68) with ejaculated sperm	TUNEL (20)	NR	TESA	NR	58.0; range: 20.0–100.0	40.0	NR
Esteves *et al*. ^24^ (2015)	Prospective cohort	Oligozoospermic (sperm concentration 5–15 million/mL) infertile men (172); couples with no history of ICSI failure (Testi-ICSI, n = 81 and Ejac-ICSI, n = 91)	SCD (30)	40.9 ± 10.2 (E) and 8.3 ± 5.3 (T) ( *P* <0.001)	TESE and TESA	100.0 and 6.2	69.4 (E) vs. 56.1 (T) ( *P* = 0.0001)	40.2 (E) vs. 51.9 (T) (NS)	LBR: 26.4 (E) vs. 46.7 (T) ( *P* = 0.007)
Mehta *et al*. ^25^ (2015)	Case series	Oligozoospermic (sperm concentration <5 million/mL) infertile men (24); couples with one or more failed IVF or ICSI cycles using ejaculated sperm	TUNEL (7)	24.0 (95% CI 19–34) (E) and 5.0 (95% CI 3–7) (T) ( *P* = 0.001)	Micro-TESE	100.0 and NR	54.0	50.0	50.0
Bradley *et al*. ^27^ (2016)	Retrospective cohort	Predominantly oligozoospermic infertile men; Testi-ICSI (n = 148) ^d^, Ejac-ICSI (n = 80) ^d^	SCIT (29)	NR	TESE and TESA	NR	66.0 (E) vs. 57.0 (T) ( *P* <0.001)	27.5 (E) vs. 49.5 (T) ( *P* <0.01)	LBR: 24.2 (E) vs. 49.8 (T) ( *P* <0.05)
Pabuccu *et al*. ^28^ (2016)	Retrospective cohort	Normozoospermic infertile men (71); couples with history of ICSI failure using ejaculated sperm (Testi-ICSI, n = 31; Ejac-ICSI, n = 40)	TUNEL (30)	41.7 ± 8.2 (E)	TESA	100.0 and NR	74.1 ± 20.7 (T) vs. 71.1 ± 26.9 (E) (NS)	41.9 (T) vs. 20.0 (E) ( *P* = 0.04)	OPR: 38.7 (T) vs. 15.0 (E) ( *P* = 0.02)
Arafa *et al*. ^29^ (2018)	Prospective cohort; interventions applied in the same patients	Oligozoospermic and normozoospermic infertile men (36); couples with history of ICSI failure performed with ejaculated sperm	SCD (30)	56.3 ± 15.3 (E)	TESA	100.0 and NR	46.4 (T) vs. 47.8 (E) (NS)	38.9 (T) vs. 13.8 (E) ( *P* <0.0001)	LBR: 38.9 (T) vs. 8.0 (E) ( *P* <0.0001)
Zhang *et al*. ^30^ (2018)	Prospective cohort ^e^	Oligozoospermic and normozoospermic infertile men (102); couples with no history of ICSI failure (Testi-ICSI, n = 61; Ejac-ICSI, n = 41)	SCSA (30)	NR	TESA	100.0 and NR	70.4 (T) vs. 75.0 (E) (NS)	36.0 (T) vs. 14.6 (E) ( *P* = 0.01)	LBR: 36.0 (T) vs. 9.8 (E) ( *P* = 0.001)
Herrero *et al*. ^31^ (2019)	Retrospective cohort	Couples with no previous live births and a history of at least two previous failed ICSI cycles with ejaculated sperm (Testi-ICSI, n = 77; Ejac-ICSI, n = 68)	SCSA (25); TUNEL (36%)	NR	TESE	NR	SCSA: 66.3 (T); 62.9 (E) (NS) TUNEL: 61.2 (T); 57.6 (E) (NS)	SCSA: 18.2 (T); 9.1% (E) ( *P* <0.02) TUNEL: 23.1 (T); 0.0 (E) ( *P* <0.02)	^f^SCSA: 21.7 (T); 9.1 (E) ( *P* <0.01) TUNEL: 20.0 (T); 0.0 (E) ( *P* <0.02)
Alharbi *et al*. ^32^ (2019)	Retrospective cohort	Couples with one or more failed ICSI cycles with ejaculated sperm Testi- ICSI, n = 52; Ejac-ICSI, n = 48)	SCSA (15); subgroup analysis using SCSA thresholds of 30%	NR	TESA	100.0 and NR	58.0 ± 27.0 (T) vs. 70.0 ± 23.0 ( *P* = 0.03)	DFI >15%: 48.6 (T) vs. 38.7 (E); DFI >30%: 48.0% vs. 25.0% ( *P* = 0.25)	^g^DFI >15%: 36.4 (T) vs. 30.0 (E); DFI >30%: 29.2 vs. 25.0 (NS)

^a^Herrero *et al*. ^31^ reported cumulative live birth rates.

^b^2PN fertilization rate with use of testicular sperm; data from previous cycles with use of ejaculated sperm not provided.

^c^The authors reported only one pregnancy with ejaculated sperm which miscarried.

^d^Number of intracytoplasmic sperm injection (ICSI) cycles.

^e^Inferred from the study’s reported data.

^f^Cumulative live birth rates.

^g^Alharbi *et al*. ^32^ reported pregnancy rates per embryo transfer; live birth data were incomplete as a number of patients achieving clinical pregnancy were lost in follow-up. E, ejaculated sperm group; Ejac-ICSI, ICSI with ejaculated sperm; LBR, live birth rate; micro-TESE, microdissection testicular sperm extraction; NR, not reported; NS, not significantly different; OPR, ongoing pregnancy rate; SCD, sperm chromatin dispersion; SCIT, sperm chromatin integrity test, a variation of sperm chromatin structure assay (SCSA); SDF, sperm DNA fragmentation; T, testicular sperm group; TESA, testicular sperm aspiration; TESE, Testicular sperm extraction, Testi-ICSI, ICSI with testicular sperm; TUNEL, terminal deoxyribonucleotide transferase–mediated dUTP nick-end labeling assay.

Recent studies providing live birth data corroborate the effectiveness of testicular sperm for ICSI in men with high SDF ^29– 31^. Thus, despite the limited evidence and lack of randomized controlled trials, data from seven retrospective studies and three prospective studies, including a total of 830 patients and 902 ICSI cycles, suggest that Testi-ICSI is superior to ICSI with ejaculated sperm to overcome infertility among non-azoospermic men with elevated SDF in semen. Testi-ICSI has been postulated to bypass post-testicular sperm chromatin damage caused by oxidative stress during sperm transit through the epididymis ^33^. As a result, the chances of oocyte fertilization by genomically intact spermatozoa and formation of a normal embryonic genome are increased, thus positively impacting the likelihood of achieving a live birth. Notably, a single study ^32^ including 110 couples with sperm DNA damage data failed to corroborate the latter findings; however, in that study, SDF thresholds of 15% (by sperm chromatin structure assay, or SCSA) were used to select couples eligible for Testi-ICSI; those thresholds are not fully consistent with the 30% SCSA thresholds reported to be associated with adverse pregnancy outcomes in ART ^34^. Thus, the inclusion of ~30% of men with SDF values between 15% and 30% in the above study might have diluted the positive effect of Testi-ICSI.

## Severe oligozoospermia and cryptozoospermia

Weissman *et al*., in 2008, reported the first series of Testi-ICSI in patients with severe oligozoospermia (<5 million sperm/mL) ^35^. The authors performed testicular sperm injections in four couples with a history of multiple failed IVF/ICSI cycles after the use of poor-quality ejaculated sperm. The male partners had sperm counts ranging from 0.2 million/mL to 2.0 million/mL. On the day of oocyte retrieval, sperm retrieval was performed, and in all cases, motile spermatozoa were retrieved from the testis. All couples achieved embryo implantation and delivery of healthy offspring after embryo transfers.

**Figure 1.**
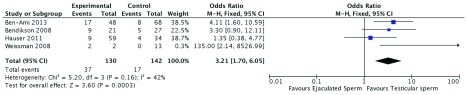
Pregnancy rates according to sperm source in non-azoospermic men with cryptozoospermia or severe oligozoospermia. Forest plot showing odds ratio for pregnancy with use of ejaculated sperm or testicular sperm for intracytoplasmic sperm injection in men with cryptozoospermia/severe oligozoospermia. CI, confidence interval; M-H, Mantel–Haenszel analysis.

Given the success reported by Weissman *et al.* ^35^, many authors sought to investigate the utility of sperm retrieval for ICSI in non-azoospermic patients with severe oligozoospermia or cryptozoospermia ( Table 2) ^36– 39^. These studies report an overall better pregnancy outcome with the use of testicular than ejaculated sperm. But surprisingly, in 2016, a systematic review and meta-analysis aggregating the data of the above studies concluded that sperm retrieval should not be recommended in men with severe oligozoospermia or cryptozoospermia ^40^. In that report, the relative risk (RR) of achieving pregnancy (272 cycles, RR = 0.53, 95% CI 0.19 to 1.42) with the use of testicular or ejaculated sperm for ICSI was not different. However, we performed a careful examination of the authors’ data and discovered that they inadvertently inverted the number of pregnancies reported in the study by Bendikson *et al.* ^36^ concerning the group of patients undergoing ICSI with testicular and ejaculated sperm. This critical mistake inflated the total number of pregnancies in the ejaculate sperm group, thus leading to an erroneous RR calculation. We reassessed the pregnancy results of the meta-analysis by Abhyankar *et al.* ^40^—after correcting the incongruency mentioned above—and found a significantly higher pregnancy rate with the use of testicular sperm than with ejaculated sperm in men with cryptozoospermia and severe oligozoospermia (272 cycles, RR = 3.21, 95% CI 1.70 to 6.05, I ^2^ = 42%, *P* = 0.0003) ( Figure 1; unpublished data).

**Table 2.** Characteristics and main outcome measures of studies reporting ICSI outcomes with testicular versus ejaculated sperm in non-azoospermic men with severe oligozoospermia/cryptozoospermia.

Study characteristics		Indication		Sperm retrieval method		Outcomes		
Author (year)	Design	Subjects and cohort size (N)	SDF assessment	Sperm retrieval method	Sperm retrieval success and complication rates (%)	Fertilization rate (%)	Clinical pregnancy rate (%)	Live birth rate (%)
Weissman *et al*. ^35^ (2008)	Case series	Severe oligozoospermic (<5 million/mL) infertile men (4) undergoing Testi-ICSI; couples with a history of multiple failed ICSI cycles with ejaculated sperm; in total, five TESA-ICSI cycles were carried out in the cohort of four patients	No	TESA	100.0 and NR	67.6	75.0	75.0
Bendikson *et al*. ^36^ (2008)	Case series	Cryptozoospermic infertile men (16); couples with history of IVF/ICSI failure (16) with ejaculated sperm; in total, 21 TESA-ICSI cycles were carried out in the cohort of 16 patients	No	Micro-TESE	100.0 and NR	51.7 (T) vs. 59.9 (E) (NS)	20.8 (E) vs. 47.4 (T) (NS)	20.8 (E) vs. 42.1 (T) (NS)
Hauser *et al*. ^37^ (2011)	Prospective cohort	Cryptozoospermic infertile men (13); in total, 93 ICSI cycles (ICSI with ejaculated sperm, n = 34; ICSI with fresh testicular sperm, n = 9; ICSI with frozen-thawed testicular sperm, n = 50) were carried out in the cohort of 13 patients	No	TESE	100.0 and NR	38.2 (E) vs. 50.0 (T, fresh) vs. 46.7 (T, frozen-thawed) ^a^ ( *P* <0.05, pairwise comparisons between T and E sperm)	14.3 (E) vs. 42.9 (T, fresh) vs. 12.8 (T, frozen- thawed) (NS)	14.3 (E) vs. 42.9 (T, fresh) vs. 12.8 (T, frozen-thawed) (NS)
Ben-Ami *et al*. ^39^ (2013)	Case series	Cryptozoospermic (17) infertile men; couples with multiple failed ICSI cycles using ejaculated sperm; in total, 116 ICSI cycles (Testi-ICSI, n = 48; Ejac-ICSI, n = 68) were carried out in the cohort of 16 patients	No	TESE	100.0 and NR	38.0 (E) vs. 46.7 (T) (NS)	15.1 (E) vs. 42.5 (T) ( *P* = 0.004)	9.4 (E) vs. 27.5 (T) ( *P* = 0.028)
Ketabchi ^41^ (2016)	Prospective cohort	Cryptozoospermic (<10 ^3^ sperm/mL) infertile men (73) undergoing ICSI with sperm retrieved from the epididymis or testis (18)	No	PESA and TESE	100.0 and NR	55.3 (E) vs. 85.7. (T+E) ( *P* <0.001)	31.6 (E) vs. 57.1 (T) ( *P* <0.001)	NR
Cui *et al*. ^42^ (2017)	Retrospective cohort	Cryptozoospermic infertile men undergoing Testi-ICSI; couples (285) undergoing ICSI with ejaculated sperm (214) or testicular sperm (71)	No	TESA and TESE	97.9 and NR	59.6 (E) vs. 60.6 (T) (NS)	33.3 (E) vs. 53.6 (T) ( *P* <0.01)	27.1 (E) vs. 44.0 (T) ( *P* = 0.03)
Yu *et al.* ^45^ (2019)	Retrospective cohort	Cryptozoospermic infertile men (35) undergoing Testi-ICSI; in total, 19 cycles (18 patients) were performed with ejaculated sperm and 19 cycles (17 patients) with testicular sperm	No	TESA and micro-TESE	100.0 and NR	74.7 (E) and 62.4 (T) in men <35 years old ( *P* = 0.01); 60.9 (E) and 56.6 (T) in men ≥35 years old (NS)	74.7 (E) and 62.4 (T) in men <35 years old ( *P* = 0.01); 60.9 (E) and 56.6 (T) in men ≥35 years old (NS)	44.4 (E) and 52.9 (T) in men <35 years old (NS); 0.0 (E) and 42.9 (T) in men ≥35 years old

^a^2PN fertilization using motile sperm. E, ejaculated sperm group; Ejac-ICSI, intracytoplasmic sperm injection with ejaculated sperm; LBR, live birth rate; micro-TESE, microdissection testicular sperm extraction; NR, not reported; NS, not significantly different; OPR, ongoing pregnancy rate; SDF, sperm DNA fragmentation; T, testicular sperm group; TESA, testicular sperm aspiration; TESE, Testicular sperm extraction, Testi-ICSI, intracytoplasmic sperm injection with testicular sperm.

Recently, additional reports and systematic reviews on the matter concerned were published ^41– 45^. In a 2018 systematic review and meta-analysis, Kang *et al*. pooled the data of six studies including a total of 578 patients and 761 ICSI cycles ^43^. The authors showed that sperm retrieval and Testi-ICSI improved the likelihood of achieving good-quality embryos (RR = 1.17, 95% CI 1.05 to 1.30, *P* = 0.005), implantation (RR = 1.52, 95% CI 1.02 to 2.26, *P* = 0.04), and pregnancy (RR = 1.74, 95% CI 1.20 to 2.52, *P* = 0.004). These results were corroborated by Ku *et al*., who pooled the evidence of studies that provided miscarriage and live birth data ^44^. The authors included a total of 331 patients and 479 ICSI cycles. In that report, miscarriage rates were not affected by the use of testicular or ejaculated sperm for ICSI (RR = 1.06, 95% CI 0.48 to 2.35), but live birth rates per initiated cycle were increased among couples that had undergone Testi-ICSI (RR = 1.77, 95% CI 1.28 to 2.44, *P* = 0.0005).

Collectively, evidence from seven retrospective studies and one prospective study, including a total of 613 patients and 799 ICSI cycles, suggests that Testi-ICSI is superior to ICSI with ejaculated sperm to overcome infertility among non-azoospermic men with severe oligozoospermia or cryptozoospermia ( Table 2). Likewise, Testi-ICSI has been postulated to bypass post-testicular sperm damage during sperm transit through the genital tract. However, no randomized controlled study has been published yet to support the routine use of sperm retrieval and testicular sperm for ICSI to non-azoospermic men with low sperm count undergoing ICSI.

## Confounding factors

The relatively low testicular sperm positivity for DNA damage might explain the better reproductive outcomes with the use of testicular sperm rather than ejaculated sperm for ICSI. Nevertheless, it is important to acknowledge that the evidence concerning the superiority of Testi-ICSI relies overwhelmingly on cohort studies with few patients, in which confounding factors, such as maternal and paternal age, etiology of male factor infertility, use of medication with possible gonadotoxic effect, and lifestyle factors, to cite a few, were not properly controlled. For instance, it has been suggested that the adverse effect of sperm DNA damage on reproductive outcomes is modulated by female age because of the intrinsic (albeit limited) capacity of oocytes from young women to repair the DNA damage ^46– 48^. On the other hand, women of advanced reproductive age have significantly fewer euploid embryos available for transfer, which will reduce ART success irrespective of the type of sperm used ^49^. Since not all sperm DNA damage is repairable, it seems sound to suggest that surgically retrieved sperm should not be used as a last resort after years of treatment with ejaculated sperm because the oocyte apparatus to repair sperm DNA damage is less efficient as both ovarian reserve and maternal age increase ^48^. These observations highlight the importance of controlling for confounders in future studies evaluating the clinical utility of testicular sperm in non-azoospermic men.

## Technical aspects

**Figure 2.**
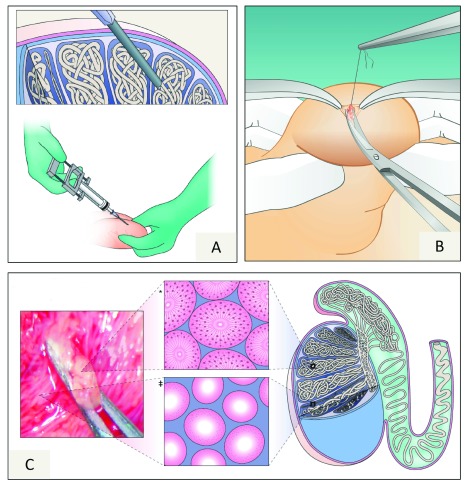
Sperm retrieval methods ( **A**) Testicular sperm aspiration. The illustration depicts a 13G needle—connected to a 20-mL syringe and fitted to the Cameco holder—being percutaneously inserted into the testis. Negative pressure is created, and the tip of the needle is moved within the testis to disrupt the seminiferous tubules and sample different areas. ( **B**) Testicular sperm extraction (TESE). Single or multiple incisions are made on the tunica albuginea, and one or several testicular biopsies are taken. ( **C**) Microsurgical TESE (micro-TESE). With aid of an operating microscope, the dilated seminiferous tubules are identified and removed with microforceps. The illustration in the middle of the figure depicts histopathology cross-sections of dilated seminiferous tubules with active spermatogenesis* and a thin tubules with germ cell aplasia ^‡^. Adapted by permission from Macmillan Publishers Ltd ^3^.

Both percutaneous and open sperm retrieval procedures can be used to harvest sperm from the seminiferous tubules in non-azoospermic men ( Figure 2) ^50– 52^. The testicle rather than the epididymis is the target organ because of the reported lower SDF rates in the former ^53– 55^. In such patients, the reported sperm retrieval success rates are close to 100% with the use of testicular sperm aspiration (TESA), testicular sperm extraction (TESE), or microdissection TESE (micro-TESE) ( Table 1 and Table 2). Our choices are TESA for men with elevated SDF and TESE or micro-TESE for cryptozoospermic patients ^24, 56^. In our hands, these methods are carried out on an outpatient basis on the same day of oocyte retrieval ^50– 52, 56– 58^. The reason relates to the fact that prolonged sperm incubation—in particular, at 37°C—and sperm freezing might negatively affect sperm chromatin integrity ^21, 59, 60^.

In the context of non-azoospermic men, sperm retrieval is associated with few complications (less than 5%) as minimal tissue extraction yields sufficient numbers of sperm for ICSI ^26, 33, 51, 52^. Nevertheless, given the potential risk for complications and adverse effects on testicular function, sperm retrieval should be performed by well-trained urologists.

## Offspring health

The use of sperm retrieval in non-azoospermic men has raised concerns about the health of resulting offspring because of the reports of increased sperm aneuploidy rates in testicular sperm (versus ejaculated sperm) ^23, 61– 64^. On the one hand, ICSI has been associated with possible increased risks of congenital malformations, epigenetic disorders, chromosomal abnormalities, infertility, cancer, delayed psychological and neurological development, and impaired cardiometabolic profile compared with naturally conceived children and this is probably due to the influence of parental subfertility ^3^. On the other hand, data concerning risks and sequelae to offspring health with the use of surgically retrieved gametes from azoospermic men are overall reassuring albeit limited ^3, 65– 70^. However, no study has yet examined whether ICSI with testicular instead of ejaculated sperm (when both are available) affects the risk of malformations and long-term health of offspring.

Nevertheless, new data generated by whole-exome sequencing molecular karyotype suggest that sperm aneuploidy in testicular specimens is not a major concern ^71^. In this series, paired assessments in ejaculated and surgically retrieved testicular samples of non-azoospermic patients with elevated SDF in semen showed that the rates of aneuploidy (1.3% versus 8.4%, respectively, *P* = 0.02) were lower in testicular sperm than in ejaculated sperm. Along these lines, Weng *et al*. showed that the origin of sperm used for ICSI had no marked influence on embryo aneuploidy rates ^72^. Moreover, a 2019 ICSI study from our group—using 24-chromosome genetic testing—revealed that euploid blastocyst rate per metaphase II oocyte was not differently affected whether ejaculated or testicular sperm retrieved from men with elevated SDF was used for ICSI (18.7% versus 18.2%, respectively) ^73^. These observations corroborate the safe utilization of sperm retrieval in non-azoospermic men, but owing to limited data concerning the health of resulting offspring, continuous monitoring is warranted.

## Conclusions

A growing body of evidence supports sperm retrieval for ICSI in non-azoospermic men with elevated SDF, severe oligozoospermia, and cryptozoospermia. In these scenarios, Testi-ICSI instead of ICSI with ejaculated sperm seems to be associated with improvements in pregnancy outcomes. Percutaneous aspiration and open TESE (with and without the aid of microsurgery) are the methods that have been applied, with high success rates and few complications, to harvest sperm from the seminiferous tubules of non-azoospermic men. However, it is essential to acknowledge the limitations of existing evidence. First, most of the data summarized derive from small observational studies in which confounder factors were not properly controlled. Thus, level 1 evidence in support of Testi-ICSI is still lacking. Second, sperm retrieval is an invasive procedure with potential complications. Thus, identification and treatment of the male factor associated with high SDF, oligozoospermia, and cryptozoospermia are essential to potentially avoid the use of surgical retrieval. We recommend a holistic approach to improve paternal health—whenever there is an opportunity—to patients embarking on any type of ART treatment. Lastly, there are limited data concerning the health of resulting offspring with the use of sperm retrieval and ICSI in cases where both ejaculated and testicular sperm are available. Keeping these limitations in mind, by summarizing the current literature, this article might guide health-care providers in presenting available evidence to patients to help them make informed decisions.
